# Remodeling of astrocyte secretome in amyotrophic lateral sclerosis: uncovering novel targets to combat astrocyte-mediated toxicity

**DOI:** 10.1186/s40035-022-00332-y

**Published:** 2022-12-26

**Authors:** Winanto Ng, Shi-Yan Ng

**Affiliations:** Institute of Molecular and Cell Biology, A*STAR Research Entities, Singapore, 138673 Singapore

**Keywords:** Motor neuron disease, Astrocyte, Astrocyte-mediated toxicity, Secretome

## Abstract

Amyotrophic lateral sclerosis (ALS) is an adult-onset paralytic disease characterized by progressive degeneration of upper and lower motor neurons in the motor cortex, brainstem and spinal cord. Motor neuron degeneration is typically caused by a combination of intrinsic neuronal (cell autonomous) defects as well as extrinsic (non-cell autonomous) factors such as astrocyte-mediated toxicity. Astrocytes are highly plastic cells that react to their microenvironment to mediate relevant responses. In neurodegeneration, astrocytes often turn reactive and in turn secrete a slew of factors to exert pro-inflammatory and neurotoxic effects. Various efforts have been carried out to characterize the diseased astrocyte secretome over the years, revealing that pro-inflammatory chemokines, cytokines and microRNAs are the main players in mediating neuronal death. As metabolomic technologies mature, these studies begin to shed light on neurotoxic metabolites such as secreted lipids. In this focused review, we will discuss changes in the astrocyte secretome during ALS. In particular, we will discuss the components of the reactive astrocyte secretome that contribute to neuronal death in ALS.

## Introduction

Amyotrophic lateral sclerosis (ALS) is an adult-onset paralytic disease characterized by progressive degeneration of upper (cortical) and lower (spinal) motor neurons from motor cortex, brainstem and spinal cord [1]. Motor neurons serve as important communication links between the brain and voluntary muscles, so the progressive neurological deterioration of motor neurons results in muscular atrophy and ultimately death from respiratory failure within 1–5 years after disease onset [2]. About 5%–10% of ALS cases are familial and the remaining 90% are sporadic [3]. Beyond motor neurons, numerous studies also support the roles of astrocyte-mediated toxicity in the onset and progression of ALS [4–6]. Astrocytes are the major glial cell type in the adult central nervous system (CNS), constituting 20%–40% of the human brain [7, 8]. Despite their abundance, the roles of astrocytes in modulating neurodegeneration are just starting to be uncovered. Astrocytes are highly secretory cells that play diverse roles in supporting neuronal health, such as modulating the blood-brain barrier [9], regulating synaptogenesis [10, 11] and neurotransmitter recycling [12], providing metabolic support to neurons [13], and regulating CNS inflammatory responses [14], which have been extensively reviewed previously [15–17]. It has also been well-documented that diseased astrocytes contribute to neuronal defects and death. Healthy neurons co-cultured with ALS astrocytes display neuronal deficits that result in cell death [6, 18–22]. In addition to reduced secretion of neurotrophic factors and metabolites that support neuronal survival and function [23], the neurotoxic effects are also considered to be mediated by secreted “toxic factors” [22] such as pro-inflammatory cytokines. ALS astrocytes are known to take on an inflammatory reactive state [24], which triggers the release of pro-inflammatory cytokines and chemokines that initiate an inflammatory cascade that results in neuronal damage and death [25]. An increasing body of evidence also suggests contribution of multiple other bioactive molecules to ALS astrocyte-mediated toxicity, such as lipids, metabolites, microRNAs and even extracellular matrix proteins [24, 26, 27].

Major challenges exist in studies of astrocyte-mediated toxicity, as the components of astrocyte secretome and their changes in disease conditions remain poorly understood. What constitute a healthy astrocyte secretome? How do the secretory profiles of astrocytes change with ALS progression? Do the changes in ALS astrocyte secretome contribute to motor neuron damage and death? In this review, we aim to shed light on some of these questions by summarizing the key findings supporting the relevance of astrocyte secretome with ALS and discuss the efforts made to elucidate components of the astrocyte secretome. Finally, we will discuss if the astrocyte secretome can be exploited for therapeutic intervention for treatment of ALS.

## Astrocytes are not static cell types, but rather adapt quickly to extrinsic stimuli

Astrocytes are highly plastic cell types, and can acquire different phenotypes in response to pathological stimuli during neurodegeneration, injury or infection [28]. In healthy nervous tissues, astrocytes are quiescent and support normal neuronal metabolism and function [13]. However, upon injury of nervous tissue caused either by trauma or neurodegeneration, astrocytes are activated through a process known as reactive astrogliosis with a change of phenotype aimed to regulate neuroinflammation [17]. Known molecular triggers of reactive astrogliosis include pro-inflammatory molecules such as tumor necrosis factor alpha (TNF-α) and IL-1β [29], which have the ability to induce changes in gene and protein levels of quiescent astrocytes, leading to secretion of neurotoxic factors.

### Reactive astrocytes are defined by their neurotoxic, proinflammatory (A1) phenotype or neuroprotective, anti-inflammatory (A2) phenotype

Astrocyte activation is a defense to CNS insults and pathologies, aimed at minimizing and repairing the damage [30]. Reactive astrogliosis is the process where astrocytes remodel their transcriptome, metabolome, secretome and morphology in response to pathology. Although reactive astrocytes have been classified into A1 and A2 phenotypes based on their respective neurotoxic or neuroprotective properties [31–35], it is increasingly recognized that such binary classifications do not fully represent the heterogeneity of reactive astrocytes [36, 37]. However, at the time of writing this review, the extent of reactive astrocyte heterogeneity in ALS is not entirely elucidated. Therefore, we will discuss changes in ALS astrocytes based on this binary A1 versus A2 astrocyte classification to explain how understanding astrocyte remodeling is important for the discovery of novel therapeutics targeting astrocyte-mediated toxicity.

A1 reactive astrocytes promote cytotoxicity in part by secreting toxic factors that result in neuronal death and demyelination [31, 32] while A2 astrocytes are neuroprotective and promote nervous system repair by upregulating pro-survival factors [33–35]. At the molecular level, A1 and A2 astrocytes differ in their gene expression signature. The A1 astrocytes are defined by expression of the complement cascade component C3 and interferon-induced guanylate-binding protein 2, and activation of the nuclear factor kappa B (NF-κB) pathway [32, 38]. On the other hand, the neuroprotective A2 astrocytes are defined by expression of S100A10, which promotes cell proliferation and membrane repair and inhibits apoptosis [32]. The A2 astrocytes also promote expression of epithelial membrane protein EMP1 [39] and anti-inflammatory cytokine transforming growth factor beta (TGF-β), which prevents synaptic loss and neuronal damage [40, 41].

Recent studies have investigated extracellular and intracellular signaling pathways which determine the A1 or A2 fate of astrocytes. Microglia and neurons are major sources of extracellular signals (such as chemokines and cytokines) that polarize astrocytes into its neurotoxic or neuroprotective state. Liddelow and colleagues have demonstrated that complement component 1 subcomponent q (C1q), interleukin-1α (IL-lα), and TNF-α secreted by lipopolysaccharide (LPS)-activated microglia can induce the A1 phenotype of astrocytes in vitro and in vivo [32]. In another study, activation of nod-like receptor family pyrin domain-containing 3 (NLRP3) by microglia was demonstrated to transform astrocytes into the cytotoxic A1 phenotype [42]. The NLRP3 inflammasomes can further process pro-IL-18 into IL-18. When added exogenously into primary astrocyte cultures, IL-18 induces downregulation of A2 astrocytic markers and upregulation of A1 neurotoxic markers [32, 43]. Ultimately, these various exogenous signals converge to the NF-κB signaling pathway, which modulates the A1 neuroinflammatory response [32, 44]. Elevated NF-κB activity has also been detected within the spinal cord astrocytes of ALS patients [45]. Interestingly, physiological aging has also been shown to induce A1-like astrocyte reactivity [39], likely due to the activated microglial cells that are formed during aging, as mice lacking the microglial-secreted cytokines IL-1α, C1q and TNF-α show low expression of A1-reactive astrocyte markers [32].

**Fig. 1:**
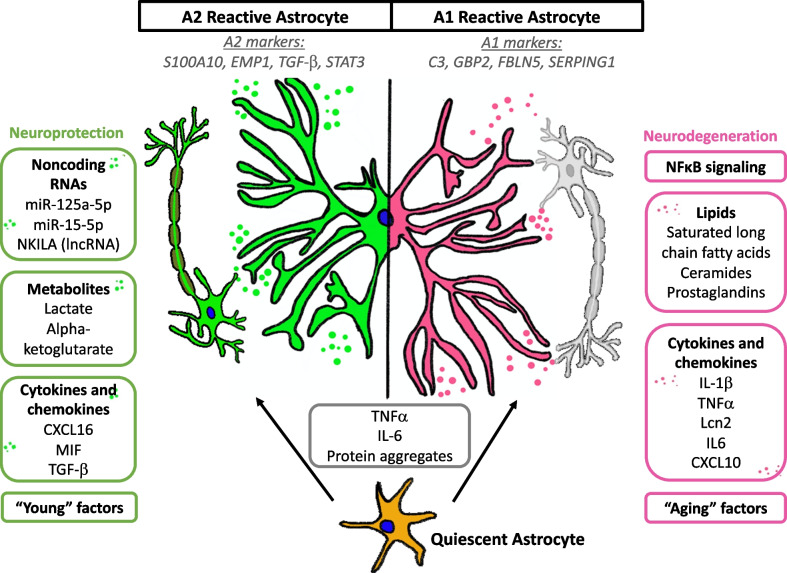
Changes in astrocyte secretome when a quiescent astrocyte takes on an A2 or A1 phenotype. A1 and A2 reactive astrocytes have distinct intracellular markers, as well as different secretory profiles. A2 reactive astrocytes promote neuroprotection through secretion of anti-inflammatory cytokines and chemokines as well as anti-apoptotic non-coding RNAs. In addition, enhanced secretion of metabolites such as lactate and alpha-ketoglutarate provides additional metabolic support for neurons. A1 reactive astrocytes, on the other hand, are neurotoxic, and promote neurodegeneration through secretion of pro-inflammatory cytokines and chemokines. Recent studies also show that these astrocytes secrete neurotoxic fatty acids and lipids, which represents a paradigm shift in the field of neurodegeneration

On the other hand, the anti-inflammatory cytokine IL-10 secreted predominantly by astrocytes and microglia has been shown to induce the neuroprotective A2 phenotype. Astrocytes derived from mice with low expression of IL-10 have elevated levels of A1-type markers as compared to wild-type mice under normal conditions or when challenged with LPS. When astrocytes are pre-treated with IL-10, the A1 transcripts are decreased and behavioral deficits are reduced, suggesting that IL-10 is an inhibitor for A1 astrogliosis [46]. Prokineticin-2 (PK2) is a secreted neuropeptide and plays a neuroprotective role [47]. Astrocytes express high levels of the PK2 receptor PKR1. It has been shown that PK2 treatment or overexpression in primary astrocyte cultures can promote the A2 astrocyte fate. Likewise, depletion of PKR1 in mice results in a decrease in A2 markers while chemical agonists of PKR1 promote the A2 neuroprotective phenotype in astrocytes [48]. A summary of A1 versus A2 reactive astrocytes is provided in Fig. 1.

### Astrocytes become reactive in response to ALS-associated misfolded proteins

A major pathological hallmark of ALS is the presence and accumulation of cytoplasmic inclusions containing TAR DNA-binding protein 43 (TDP43), FUS or superoxide dismutase 1 (SOD1) protein [49–52] in neurons and astrocytes. Studies suggest that these protein aggregates form insoluble fibrils that may propagate in a prion-like manner where the fibrils act as seeds to trigger aggregation of native counterparts [52, 53]. Sequestration of native proteins such as TDP43, FUS and SOD1 into insoluble aggregates within the cytosol would effectively mimic a loss-of-function mutation since the mislocalization and the sequestration of the native proteins imply that their physiological activities are compromised [54, 55]. Astrocytic depletion of TDP43 has been shown to cause reactive astrogliosis with a pro-inflammatory phenotype [56, 57]. Notably, mice with selective TDP43 deletion in astrocytes (*GFAP-Cre; tardbp*^*fl/fl*^ mice) have enhanced GFAP immunoreactivity with longer astrocytic processes along with increased C1q expression, indicative of astrogliosis. Additionally, RNA sequencing of these TDP43-deficient astrocytes reveals a molecular signature of pro-inflammatory A1 reactive astrocytes [57].

Insoluble fibrils themselves may also be triggers of astrogliosis, which has been demonstrated in other adult-onset neurodegenerative diseases such as Parkinson’s disease that fibrillar alpha-synuclein species induce neurotoxic A1-type astrogliosis via NFκB activation [58]. In the context of ALS, overexpression of TDP43 in astrocytes, which likely results in cytoplasmic aggregation [59], induces secretion of pro-inflammatory cytokines (such as TNF-α and IL-6) and causes neurotoxicity in a neuron-astrocyte coculture system [60].

There is also accumulating evidence suggesting that soluble oligomeric forms of protein aggregates are responsible for neurotoxicity in diseases where protein misfolding is a prominent feature. For instance, in Alzheimer’s disease, oligomeric forms of amyloid-beta induce high levels of pro-inflammatory molecules such as IL-1β, inducible nitric oxide synthase, NO and TNF-α in cultured rat astrocytes, consistent with an A1-reactive phenotype [61]. In ALS, TDP43 has been shown to be capable of forming oligomeric protofibrils [62, 63]. Exogenous TDP43 oligomers are found to be toxic to motor neurons but not to astrocytes [64]. Since Patani and colleagues showed that TDP43 oligomers trigger aggregation in astrocyte cultures, it is plausible that they would also lead to astrogliosis, although there are currently no available transcriptomic or protein expression data to confirm this. Apart from TDP43, mutant SOD1 can also form corkscrew oligomers which result in axonopathies and mitochondria defects, two hallmarks of ALS-linked pathologies [65].

Within the spinal cord, reactive astrocytes induce motor neuron pathologies similar to that seen in ALS [66], where proteostasis and autophagy are dysregulated in motor neurons, which eventually result in their selective death [67]. Altogether, these various studies highlight that astrocytes respond to various stimuli, including factors secreted by microglia and neurons, affecting the balance between A1 and A2 reactive astrogliosis. In ALS patients, astrocytes display the A1-type characteristics and are major players in motor neuron degeneration [19]. Corroborating this finding, Ziff and colleagues performed a systemic meta-analysis of publicly-available sequencing data of ALS astrocytes, which included human induced pluripotent stem cell-derived astrocytes and mouse primary astrocytes carrying various ALS-associated mutations. They concluded that ALS astrocytes are characterized by an “A1-like” inflammatory reactive signature, defined by upregulation of genes involved in extracellular matrix remodeling, cellular stress and immune response, while expression of genes involved in astrocyte functions such as glutamate uptake and other neuronal support mechanisms is suppressed [24].

### Astrocytes respond to neurotransmitters by modulating endocytosis, exocytosis, and survival

In addition to their immunomodulatory roles, astrocytes also play a role in neurotransmitter recycling. Excitotoxicity is thought to be a major contributor to motor neuron degeneration in ALS [68–70]. Riluzole, a food and drug administration (FDA)-approved drug for ALS, works by blocking excitotoxicity through inhibiting glutamatergic neurotransmission in the central nervous system [71, 72]. Edaravone and AMX0035 are two other FDA-approved drugs for treatment of ALS. Edaravone works as a reactive oxygen species (ROS) scavenger [73]. AMX0035 is a combination of two drugs phenylbutyrate and tauroursodeoxycholic acid, and works by inhibiting endoplasmic reticulum stress response and blocking mitochondria-mediated apoptosis and ROS production [74–76]. Of the three approved drugs, only Riluzole works by suppressing excitotoxicity and therefore reducing astrocyte toxicity.

Glutamate is the most abundant excitatory neurotransmitter in the nervous system and is found with high concentrations in synaptic vesicles in pre-synaptic nerve terminals where it is released by exocytosis. After exocytosis, glutamate binds to several receptors at the post-synaptic membrane including *N*-methyl-*D*-aspartate (NMDA), α-amino-3-hydroxy-5-methy-4-isoxazolepropionic acid (AMPA), kainate and mGluR receptors. Excitotoxicity occurs when neurons are exposed to high levels of glutamate, which results in persistent activation of NMDA receptor, AMPA receptor, and voltage-gated calcium channels, and consequently a lethal influx of extracellular calcium [77]. Therefore, rapid glutamate clearance at the synaptic cleft is necessary and this process is mediated by glutamate transporters or excitatory amino acid transporters (EAATs). Five EAATs have been identified to date (EAAT-1 to EAAT-5), of which EAAT-1 and EAAT-2 are primarily expressed on astrocytes [78]. Notably, EAAT-2 expression is altered in ALS, which may contribute to the excessive levels of glutamate in the cerebrospinal fluid in patients [70, 79, 80]. In astrocytes, glutamate is converted into glutamine by the enzyme glutamine synthetase, and is released back into the extracellular space, where it will be taken up by neurons to produce glutamate. This process is known as the glutamate-glutamine cycle.

High levels of glutamate can cause astrocyte depolarization [81, 82], which leads to the exocytosis of gliotransmitters such as glutamate, ATP and neurotrophic factors and less commonly, *D*-serine, adenosine and prostaglandins [83]. In ALS patients, high levels of cerebrospinal fluid (CSF) glutamate have been found compared to the control population, and they correlate with more severe motor impairment [84]. In ALS mice overexpressing human *SOD1*^G93A^, exposure to glutamate results in focal degeneration of spinal cord astrocytes, which is not observed in mice overexpressing wild-type *SOD1* [85]. This selective vulnerability to glutamate is caused by mGluR5 activation, as a selective mGluR5 antagonist blocks the glutamate toxicity in ALS astrocytes [85].

Glutamate toxicity is of high relevance in ALS, and contributes to ALS pathology via multiple pathways, exerting effects directly on motor neurons as well as on astrocytes, modulating the astrocytic exocytosis of gliotransmitters, thus providing paracrine signals on neighboring neurons.

## Astrocytes remodel their secretome in ALS

It is increasingly evident that ALS astrocytes contribute to neuronal dysfunction and death [18]. A number of landmark papers have demonstrated that co-culture of healthy motor neurons with ALS astrocytes or exposing them to ALS astrocyte-conditioned media results in motor neuron death [6, 86]. This suggests that ALS astrocytes secrete soluble factors that are toxic to motor neurons. Interestingly, Birger et al. [87] demonstrated that the cytotoxicity of patient-derived mutant astrocytes is positively correlated with their duration in culture, implying that aged astrocytes are more neurotoxic and this is consistent with the fact that ALS is an age-onset neurodegenerative disease. Mass spectrometry experiment comparing conditioned medium (CM) of ALS astrocytes to that of healthy control revealed up-regulation of extracellular matrix proteins (collagen, lumican, olfactomedin-like protein 3 and protein-lysine 6 oxidase) and also downregulation of antioxidant proteins (SOD1, SOD2, glutathione synthetase) and motor neuron pro-survival factors (miR-494-3p), resulting in elevated stress and senescence in both astrocytes and motor neurons [87, 88]. In another study, Skorupa et al. [89] used quantitative proteomics to profile astrocytic secretome with angiogenin exposure. Angiogenin is a neuroprotective factor secreted by motor neurons and it modulates astrocytic secretome after being taken up by astrocytes. Loss-of-function mutation of this gene has been documented in ALS patients. Astrocytes exposed to angiogenin have also been demonstrated to express significantly different levels of chemokines, cytokines, proteases, and ECM proteins.

### Astrocyte-secreted cytokines, chemokines and complements act as immunomodulatory molecules

ALS astrocytes are also known to secrete chemokines, complements and cytokines [33]. Of them, TNF-α is known to play a major role in motor neuron toxicity as neutralizing antibodies for TNF-α can partly rescue motor neuron death when co-cultured with ALS astrocytes [90]. This is not surprising given the role of TNF-α in promoting reactive astrogliosis and the A1 phenotype of astrocytes. Although the complete list of A1 astrocyte-secreted toxic factors has not been worked out, some pro-inflammatory cytokines and chemokines have been demonstrated to result in poor prognosis of ALS. In a recent study, Tortelli and colleagues reported that a panel of cytokines (IL-2, IL-6, IL-10, interferon-gamma [IFN-γ], and TNF-α) is elevated in plasma of ALS patients, with IL-6 having the highest discriminatory power between patients and control [91]. Also, a post-mortem study found changes in expression of both chemokines and cytokines in astrocytes from patients with familial amyotrophic lateral sclerosis (fALS) and sporadic amyotrophic lateral sclerosis (sALS), including upregulated chemokine (C-C motif) ligand (CCL) 2, CCL11, CCL13, CCL20, chemokine (C-X-C motif) ligand (CXCL) 1, CXCL2, CXCL3, CXCL5, CXCL6, CXCL10, CXCL11, and CXCL12, all of which are involved in the regulation of inflammation [6, 92]. These pro-inflammatory cytokines and chemokines secreted by A1-subtype ALS astrocytes can lead to activation of the NF-κB signaling, and prolonged activation of this inflammatory pathway would lead to accelerated disease progression and eventual collapse of the blood-spinal cord barrier [93].

At present, the exact mechanisms of how astrocyte-mediated neuroinflammation leads to motor neuron death in ALS still remain to be fully investigated. Apart from inducing neuroinflammation, the secreted cytokines such as TGF-β1 cause cellular toxicity through dysregulation of autophagy, resulting in aberrant protein aggregation [67]. Furthermore, A1 astrocytes also secrete IL-6, IFN-γ and prostaglandins, which, together with increased ROS, glutamate and nitric oxide, lead to reduction of neurotrophic factors such as vascular endothelial growth factor, brain-derived neurotrophic factor and ultimately neuronal degeneration [51, 94]. In recent years, efforts have been made to target neuroinflammation in ALS by altering the astrocytic secretome. Izrael and colleagues demonstrated a therapeutic effect of injecting “young” astrocytes derived from embryonic stem cells in ALS mice [95]. These young astrocytes secrete pro-survival factors and remodel the extracellular matrix to support neuronal growth. Specifically, young astrocytes behave as protective A2 astrocytes secreting neuroprotective chemokines such as CXCL16 and macrophage migration inhibitory factor (MIF) [39]. CXCL16 has been shown to protect neurons from excitotoxicity cell death [96] and MIF functions as chaperone preventing ALS motor neurons from degeneration caused by misfolded SOD1 [97]. Removing factors known to induce astrogliosis has been shown to improve the survival of ALS mice. Guttenplan and colleagues performed triple knockout of IL-1α, TNFα, and C1q and observed a reduction in astrogliosis and longest extension of lifespan ever reported in SOD1^G93A^ mice [98]. Collectively, ALS astrocytes play a role in the progression of disease by secreting cytokines for neuroinflammation and protein aggregation, and there is accumulating evidence suggesting that altering the secretory profiles of astrocytes can delay the progression of ALS [26, 87, 99].

### Astrocyte-secreted lipids are gaining attention as a novel class of neurotoxic molecules in ALS

In pathological conditions, lipids are a class of bioactive macromolecules that are secreted by astrocytes as potential toxic factors. Lipid droplets act as a store for excessive fatty acids and their formation can be induced by cellular stress such as hypoxia and starvation. Disrupted astrocytic lipid metabolism has also been linked to ALS. Astrocytes expressing mutant TDP43 exhibit higher accumulation of lipid droplets [100], suggesting an imbalance between lipid biosynthesis or uptake by ALS astrocytes and catabolism. Polyunsaturated fatty acids, in particular arachidonic acid, are also mediators of neurodegeneration in ALS, and are found at high levels in ALS spinal cords and cerebrospinal fluid samples [101–103]. These polyunsaturated fatty acids are possibly produced and secreted by astrocytes [104, 105], although some neurons are also known to release arachidonic acid upon depolarization [106, 107].

Arachidonic acid also serves as a precursor for prostaglandin E2 (PGE2), an eicosanoid that acts as a potent inflammatory mediator which contributes to neuroinflammation and motor neuron death. Elevated levels of PGE2 are a signature of ALS, with a majority of ALS patients having up to ten-fold higher levels of PGE2 in the CSF [108, 109]. Cyclo-oxygenase 2 (COX2) catalyzes the conversion of arachidonic acid to PGE2 [110]. Pharmacological inhibition of COX2 by specific inhibitors such as celecoxib or rofecoxib delays the development and progression of ALS by suppressing excitotoxicity, promoting the survival of motor neurons and reducing astrogliosis [111, 112].

Astrocyte-secreted long-chain saturated free fatty acids have also been implicated in neurotoxicity [113]. Fractionation of reactive-astrocyte conditioned media using biochemical purification columns revealed that hydrophobic and charged components contribute most significantly to oligodendrocyte death. The lipids bound to APOE and APOJ lipoproteins mediate the toxicity. Unbiased lipidomics of more than 1500 lipids from 10 classes revealed a significant upregulation of long-chain saturated free fatty acids in the reactive-astrocyte conditioned media. To investigate if these saturated free fatty acids are the mediators of neurotoxicity, Liddelow and colleagues generated astrocyte-specific elongation of very long chain fatty acids protein 1 (ELOVL1) conditional knockout (*Elovl1* cKO) mice, as ELOVL1 is the enzyme that catalyzes the synthesis of long-chain (more than 16 carbons), saturated lipids and its expression is upregulated in reactive astrocytes. Reactive-astrocyte conditioned media from *Elovl1* cKO mice are less toxic than that of wild-type mice, confirming the lipidomics findings [113]. In the context of ALS, enhanced expression or activity of astrocytic ELOVL1 or elevated production of saturated long-chain free fatty acids, has been reported, which warrants further investigations.

Astrocytes are the primary cell type that produces cholesterol in the adult CNS, and the production is regulated by the transcription factor sterol regulatory element binding protein-2 (SREBP2). Dodge et al. showed that overexpression of SREBP2 in the CNS results in accumulation of cholesterol and neutral lipids, as well as ALS-like symptoms in mice, such as progressive hindlimb paralysis, spasticity and reduced lifespan, suggesting that accumulation of neutral lipids is associated with spinal neuron degeneration [114]. Consistently, SOD1-G93A rodents display increased levels of lipid droplets and severe astrogliosis in the late symptomatic stage [101]. Additionally, lipids such as sphingolipids, ceramides and cholesterol are found in higher levels in ALS spinal cords [101]. However, it remains unclear if these neurotoxic lipids are primarily secreted by astrocytes or other neural cells in the spinal cord.

### Extracellular vesicles contain cargos and may reflect disease status in ALS

As highly secretory cells, astrocytes release membrane-bound vesicles or extracellular vesicles (EVs). These astrocyte-derived EVs (ADEVs) are membrane-bound vesicles released by astrocytes into the extracellular space and include exosomes and microvesicles. More importantly, ADEVs carry cargos such as peptides, nucleic acids and lipids that are key signaling entities modulating neuronal function, survival and regeneration [115]. In the healthy CNS, ADEVs play a neuroprotective role by promoting dendritic growth, survival and electrophysiological activities of neurons [116, 117]. However, under pathological conditions, reactive ADEVs accelerate disease progression and aggravate neuroinflammation [118], indicating key alterations of the cargos contained within these vesicles. To characterize the protein components of ADEVs from control and activated astrocytes, You et al. [119] first treated primary human astrocytes with IL-1β to induce astrogliosis, and performed label-free mass spectrometry to identify changes in the composition of reactive ADEVs. They found that ADEVs released by astrocytes in response to IL-1β impair neuronal functions, resulting in shorter neurite lengths and reduced neuronal firing. Mechanistically, this can be attributed to the reactive ADEV peptides associated with cellular metabolism, migration and inflammatory response. Additionally, a separate study aimed at elucidating microRNA (miRNA) cargo changes upon astrocyte activation found that reactive astrocytes triggered by stimulation with either TNFα or IL-1β release higher levels of miR-125a-5p and miR-15-5p, which target the neurotrophic tyrosine kinase receptor NTKR3 or TrkC. Upon binding to its ligand neurotrophin-3, TrkC autophosphorylates and activates downstream signaling pathways that regulate synaptic development and expression of pro-survival factor Bcl-2 [117].

Elevated levels of the pro-inflammatory cytokine IL-6 have been also detected in ADEVs derived from sporadic ALS patients [120], suggesting that cytokines can be packaged into EVs to mediate astrocyte–neuron signaling in ALS. ALS astrocytes are also known to package pathogenic proteins such as misfolded SOD1 and TDP43 into EVs, contributing to the propagation of ALS pathology in the CNS [121, 122]. A study that profiled microRNAs in ADEVs isolated from ALS patients carrying the *C9ORF72* mutations revealed that the downregulation of miR-494-3p in ALS ADEVs results in decreased axonal maintenance and motor neuron survival [123]. Although emerging studies suggest that ADEVs and their cargos are responsible for the progression of ALS, the exact compositions of ADEVs and changes of ADEV contents are still not completely understood.

## Discussion: multi-omics approaches to elucidating the astrocyte secretome

**Fig. 2:**
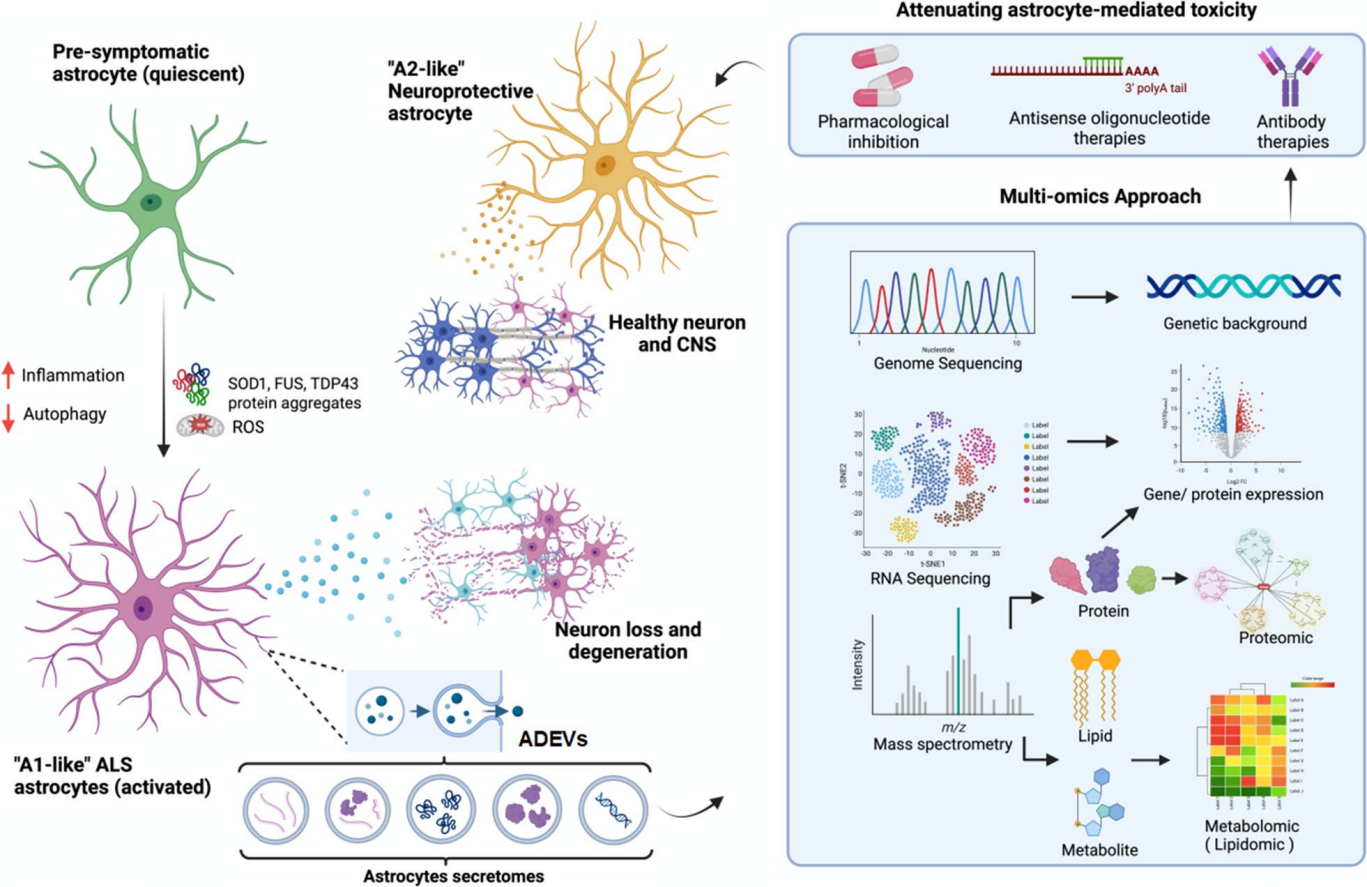
Multi-omics approaches to attenuating astrocyte-mediated toxicity. Under pre-symptomatic conditions, astrocytes play a supporting role in maintaining normal functioning of neurons and the central nervous system. As reactive oxygen species (ROS), inflammation and protein aggregates begin to accumulate, coupled with decreased ability to clear waste (autophagy), quiescent astrocytes may be activated to become ALS astrocytes which are highly secretive. These astrocyte-derived extracellular vesicles contain harmful proinflammatory cytokines and chemokines which ultimately result in degeneration of motor neurons and dysfunctional motor unit. The use of multi-omics (e.g., genomics, transcriptomics, proteomics, metabolomics) will allow for in-depth understanding of complex and multifactorial diseases such as ALS. Integration of different omics layers is crucial for uncovering changes in ALS astrocytes during disease onset and progression, paving the way for development of new therapeutics which can revert A1-like ALS astrocytes to A2 neuroprotective astrocytes

Given the vital role astrocyte-secreted factors play in maintenance of neuronal health and disease progression, it is important to identify key astrocyte targets that can complement drug development efforts for neurodegenerative diseases (Fig. 2), which tend to be heavily focused on cell-autonomous pathways in neurons. Currently, the comprehensive catalog of astrocyte-secreted factors remains to be revealed. Advancement in proteomics and deep sequencing technologies in the past decade has accelerated the identification of proteins and RNA molecules secreted by healthy and diseased astrocytes [89, 124–127]. Emerging evidence suggests that astrocyte-secreted metabolites and lipid molecules are also crucial factors that mediate neuronal health and function [128–130]. One of the most well-characterized metabolites secreted by astrocytes is lactate, the end-product of anaerobic glycolysis [131]. In the CNS, lactate is mainly produced by astrocytes, and released to be taken up by surrounding neurons at metabolic needs [132]. In the astrocyte-neuron lactate shuttle hypothesis, electrically-active neurons release the neurotransmitter glutamate, which is mainly taken up by astrocytes through glutamate transporters on their plasma membranes. This glutamatergic activation then leads to increased astrocytic glycolysis and release of lactate in the extracellular space [133]. The extracellular lactate can be transported into neurons through monocarboxylate transporter 2 to sustain neuronal activity [134]. Although this hypothesis is still debatable [133], there is increasing evidence that astrocytes provide metabolites including lactate to neurons for their various metabolic needs and function [135, 136], and changes in these metabolites can contribute to neuronal dysfunction and death.

Reactive astrocytes undergo metabolic transformations during neurodegeneration [137]. For instance, in Huntington’s disease, striatal astrocytes switch from glycolysis to fatty acid oxidation [138], while in ALS, lipid metabolism is impaired as manifested by accumulation of lipid droplets [139]. As many metabolites can become secreted, changes in metabolism during astrogliosis may lead to corresponding alterations in secretome. While proteomics and transcriptomics studies have largely elucidated the peptide and RNA components of astrocyte secretome in healthy and diseased conditions, emerging metabolomics and lipidomics studies have shown that metabolites and lipids are also relevant to astrocyte biology.

### Proteomics shows that cytokines, chemokines and neurotrophic factors are the most abundant proteins secreted by ALS astrocytes

Secreted proteins and peptides such as neurotrophic factors, anti-inflammatory cytokines (such as TGFβ and IL-10), pro-inflammatory cytokines and chemokines, and extracellular matrix proteins are thought to be a major class of functional biomolecules secreted by astrocytes [140], and many of these factors are known to alter neuronal function and activity. To elucidate secreted proteins associated with diseased astrocytes, Choi and colleagues [92] harvested human fetal astrocytes, stimulated them with IL-1β and TNF-α to mimic reactive astrogliosis, and collected their conditioned media for profiling in comparison to unstimulated astrocytes. Results revealed that stimulated and unstimulated astrocytes secrete distinct sets of cytokines and chemokines. Most of the cytokines and chemokines produced by non-stimulated and activated astrocytes are direct targets of NF-κB that plays immunomodulatory roles in the CNS. In another study relevant to neurodegeneration, conditioned media collected from spinal astrocytes derived from ALS transgenic mice overexpressing the human *SOD1*-G93A mutant were compared to wild-type astrocyte condition media using quantitative proteomics. Similar to previous report of activation of NF-κB and pro-inflammatory pathways, ALS astrocytes are shown to be depleted of metabolites involved in glutathione metabolism that plays a crucial role in redox signaling [141].

Across various disease conditions, reactive astrocytes consistently produce and secrete lower levels of neuroprotective neurotrophic factors while releasing higher amounts of neurotoxic cytokines and chemokines [33]. Additionally, other secreted factors have also been identified to play a role in neurodegeneration. Lipocalin-2 (Lcn2) secretion by astrocytes can be triggered by hypoxia [142], neurodegeneration [143–145], traumatic brain injury [146, 147] or spinal cord injury [148, 149]. Elevated Lcn2 levels have been found in the motor cortex, spinal cord, and plasma samples of post-mortem ALS patients. The secreted Lcn2 results in selective degeneration of neurons, by upregulating pro-apoptotic factor Bim and disrupting iron metabolism and inflammatory gene expression [145]. Importantly, Lcn2 shows promise as a therapeutic target. In wild-type mice, reactive astrocytes show robust expression of pro-inflammatory cytokines, such as IL-6 and CXCL10, which is attenuated in Lcn2-deficient mice [142, 145]. As a result, Lcn2 depletion promotes cell survival by reducing neuroinflammation, suggesting that manipulation of Lcn2 levels could regulate the progression of neurodegeneration.

### Transcriptomics reveals disease-associated non-coding RNAs (ncRNAs) in ADEVs

Meanwhile, it is increasingly recognized that astrocytes also secrete ncRNAs that are packaged within extracellular vesicles known as ADEVs. These ncRNAs, which include miRNAs and long non-coding RNAs (lncRNAs), have been reported to contribute to neurodegeneration, promoting astrogliosis [150] and degeneration of neuronal networks [123]. Deep sequencing approaches have accelerated the identification of RNA species that exist in ADEVs. In an earlier section, we have discussed how deep sequencing of small RNAs has identified changes within the miRNA content in ADEVs in response to astrogliosis [117]. Disease-specific miRNAs have also been described for ALS [151, 152], traumatic brain injury and spinal cord injury [153, 154], and some of them have been identified as potential therapeutic targets because abrogation of them slows down the disease progression [155–157].

The lncRNAs, defined as noncoding transcripts longer than 200 nucleotides, are highly expressed in the brain. Many of these lncRNAs are bioactive molecules that play a role in modulating gene expression and signaling pathways [158, 159]. In traumatic brain injury, astrocytes show increased expression and secretion of the lncRNA NKILA, which exerts neuroprotective properties by depleting miR-195, an miRNA associated with apoptosis [160]. However, despite the large quantity of lncRNAs expressed by astrocytes [161], comprehensive studies of astrocyte-secreted lncRNAs in disease conditions are still needed.

### Metabolomic-based studies will yield important information on disease-relevant changes of astrocyte-secreted metabolites and lipids

Astrocytes store energy in forms of glycogen and lipid droplets and mobilize metabolic stores to support neuronal function. While astrocytes normally produce ATP through complete glycolysis, they can also switch to fatty acid oxidation to supplement their energy requirements, especially in neurodegenerative conditions such as Huntington’s disease and ALS [138]. As fatty acid oxidation involves breaking down of fatty acids into acetyl-coA units to fuel the tricarboxylic acid cycle and mitochondrial respiration, lipid droplets are considered to be an important energy reservoir in astrocytes.

Defects in lipid metabolism have been linked to ALS [162–164]. Since lipids can be secreted by astrocytes, it can be expected that changes in lipid metabolism would alter the lipid secretory profile of diseased astrocytes. Blasco and colleagues in 2017 compared lipid content of CSF between ALS patients and neurotypical controls, and found a unique lipidomic signature in ALS, characterized by high levels of phosphotidylcholine, ceramides and glucosylceramides [165]. Since the CSF surrounds the brain and the spinal cord where astrocytes make up the majority of cells, it can be inferred that astrocytes contribute to the lipidomic signature in ALS CSF. Indeed, other independent studies have detected ceramides in ADEVs [166], suggesting that ceramides and other lipids highly secreted by ALS astrocytes may contribute to the astrocyte-mediated toxicity.

## Conclusion

Studies on neurodegeneration tend to be neuro-centric, where research aims to elucidate the intrinsic dysfunctions of neuronal pathways and functions that precede neuronal death. Recent studies have shown that the non-cell autonomous contributions to neurodegeneration are not negligible. Astrocytes play a more critical role in governing the health of the central nervous system than previously appreciated. They are highly responsive to environmental cues such as pro-inflammatory cytokines and chemokines, and adjust their secretome accordingly to produce either neuroprotective or neurotoxic effects (Fig. 1). Understanding the astrocyte secretome is key to dissecting molecular mechanisms underlying the progression of neurological disorders. To do so, a multi-omics approach is needed. While proteomics and transcriptomics studies have been carried out, key information is still lacking. When do A2 astrocytes turn reactive and is this process reversible? Are lncRNAs abundantly secreted by astrocytes and what roles do they play in ALS pathogenesis and progression? What metabolites and lipid molecules are secreted by astrocytes and how do they change in ALS? Can changes in metabolites be used as diagnostic biomarkers for early diagnosis of ALS? Are there neurotoxic amino acids or lipid species that compromise the function and survival of motor neurons? In this multi-omics era, we envision that characterization of diseased astrocyte secretome by proteomics, transcriptomics, metabolomics and lipidomics approaches would reveal novel insights into the disease progression as well as uncovering new disease targets, for ALS and therapeutic development (Fig. 2).

## Data Availability

References’ information used in this manuscript will be available on demand.

## Abbreviations

ADEV: Astrocyte-derived extracellular vesicle
ALS: Amytrophic lateral sclerosis
AMPA: α-amino-3-hydroxy-5-methy-4-isoxazolepropionic acid
C1q: Complement component 1q
CCL: Chemokine (C-C motif) ligand
CNS: Central nervous system
CSF: Cerebrospinal fluid
CXCL: Chemokine (C-X-C motif) ligand
CXCR: C-X-C chemokine receptor
EAAT: Excitatory amino acid transporter
ELOVL1: Elongation of very long chain fatty acids protein 1
EV: Extracellular vesicles
IFN-γ: Interferon-gamma
IL: Interleukin
LPS: Lipopolysaccharide
MIF: Macrophage migration inhibitory factor
NF-κB: Nuclear factor kappa B
NLRP3: NOD-, LRR- and pyrin domain-containing protein 3
NMDA: *N*-methyl-*D*-aspartate
PGE2: Prostaglandin E2
PK-2: Prokineticin-2
SOD: Superoxide dismutase
SREBP2: Sterol regulatory element-binding protein 2
TDP43: TAR DNA-binding protein 43
TGF-β: Transforming growth factor beta
Trk: Tropomyosin receptor kinase
TNF-α: Tumor necrosis factor alpha
